# Understanding machine learning applications in dementia research and clinical practice: a review for biomedical scientists and clinicians

**DOI:** 10.1186/s13195-024-01540-6

**Published:** 2024-08-01

**Authors:** Yihan Wang, Shu Liu, Alanna G. Spiteri, Andrew Liem Hieu Huynh, Chenyin Chu, Colin L. Masters, Benjamin Goudey, Yijun Pan, Liang Jin

**Affiliations:** 1 The Florey Institute of Neuroscience and Mental Health, 30 Royal Parade, Parkville, VIC 3052 Australia; 2 Florey Department of Neuroscience and Mental Health, The University of Melbourne, 30 Royal Parade, Parkville, VIC 3052 Australia; 3 The ARC Training Centre in Cognitive Computing for Medical Technologies, The University of Melbourne, Carlton, VIC 3010 Australia; 4 Department of Aged Care, Austin Health, Heidelberg, VIC 3084 Australia; 5 Department of Medicine, Austin Health, University of Melbourne, Heidelberg, VIC 3084 Australia

**Keywords:** Alzheimer’s dementia, Alzheimer’s disease, Dementia subtyping, Diagnosis, Disease progression, Machine learning

## Abstract

Several (inter)national longitudinal dementia observational datasets encompassing demographic information, neuroimaging, biomarkers, neuropsychological evaluations, and muti-omics data, have ushered in a new era of potential for integrating machine learning (ML) into dementia research and clinical practice. ML, with its proficiency in handling multi-modal and high-dimensional data, has emerged as an innovative technique to facilitate early diagnosis, differential diagnosis, and to predict onset and progression of mild cognitive impairment and dementia. In this review, we evaluate current and potential applications of ML, including its history in dementia research, how it compares to traditional statistics, the types of datasets it uses and the general workflow. Moreover, we identify the technical barriers and challenges of ML implementations in clinical practice. Overall, this review provides a comprehensive understanding of ML with non-technical explanations for broader accessibility to biomedical scientists and clinicians.

**Supplementary Information:**

The online version contains supplementary material available at 10.1186/s13195-024-01540-6.

## Introduction

Alzheimer’s disease (AD), the major cause of dementia, is a progressive neurodegenerative disorder that predominantly affects older people [1]. The accumulation of amyloid-beta (Aβ) and formation of neurofibrillary tangles marked by tau phosphorylation in the brain are the key hallmarks of AD [1]. Clinically, the disease can be divided into three stages: 1) preclinical AD i.e., cognitive unimpaired (CU) people with amyloid accumulation in the brain, 2) prodromal or mild cognitive impairment (MCI) and 3) Alzheimer’s dementia (ADem) [1]. This disease trajectory can vary between individuals, and preclinical AD can occur 15–20 years prior to ADem [1].

Observational longitudinal dementia datasets have been collected in diverse age groups across several (inter)national dementia cohorts (Table 1), providing rich information that enhances the granularity and scope of data science research. These datasets encompass a broad spectrum of information including biomarkers, genetics, neuropsychological evaluations, neuroimaging, omics, etc. (Table 2). Traditional statistical methods, constrained by rigid assumptions and a limited ability to handle complex interactions have shown limitations in processing these multi-modal datasets, prompting an exploration of more adaptive and comprehensive techniques such as machine learning (ML) [2]. ML is a class of algorithms that enable computers to analyze data and make decisions by identifying patterns specific to tasks [3]. These techniques can detect subtle patterns and trends in large datasets, significantly enhancing the effectiveness and productivity of data-driven research. In addition, ML has already proven successful in tracking disease, including market-ready products (e.g., Vivid E80 [4]) and FDA-approved devices (e.g., Apple's Atrial Fibrillation History Feature [5]).

**Table 1:** Major longitudinal datasets used in ML-dementia

	**Dataset**	**Participants**	**Year**	**Region**	**Follow-ups**	**Age**	**Data type**	**Focus/Aim**
1	Framingham Heart Study [6]	15,000 +	1948-present	USA	Every 2–8 years (vary by cohort)	13–62 at baseline (vary by cohort)	Demographic; Genetics; Lifestyle; Medical history	Cardiovascular and chronic diseases
2	BLSA [7]	3,000 +	1958-present	USA	Every 1–4 years (vary by age groups)	20–80 +	Cognitive assessments; Demographic; Genetics; Lifestyle; Medical history	Aging
3	Rotterdam Study [8]	15,000 +	1990-present	Netherlands	Variable	45 +	Biomarkers; Clinical; Cognitive assessments; Demographic; Genetic; Lifestyle	Cardiovascular, neurological, ophthalmological and endocrine diseases
4	ROSMAP [9]	3,000 +	1997-present	USA	Every 12 months	65 +	Cognitive assessments; Demographic; Genetics; Metabolomics; Imaging; Physical activity; Proteomics	Aging, Alzheimer’s disease
5	CLHLS [10]	80,000 +	1998-present	China	Variable	65 +	Biomarkers; Clinical; Cognitive assessments; Demographic; Lifestyle; Physical activity	Healthy longevity
6	NACC [11]	40,000 +	1999-present	USA	Every 12 months	19 +	Biomarker; Cognitive assessments; Demographic; Genetics; Imaging	Dementia
7	WRAP [12]	1,500 +	2001-present	USA	Every 24 months	40–65 at baseline	Biomarker; Cognitive assessments; Demographic; Genetics; Lifestyle; Metabolomics; Imaging	Alzheimer’s disease
8	ADNI [13]	2,000 +	2004-present	USA and Canada	Vary by phase	55–95	Biomarker; Cognitive assessments; Demographic; Imaging; Omics	Alzheimer’s disease
9	AIBL [14]	3,000 +	2006-present	Australia	Every 12–18 months	50 +	Biomarker; Cognitive assessments; Demographic; Imaging; Omics	Alzheimer’s disease
10	UK Biobank [15]	500,000 +	2006-present	UK	Variable	40–69 at baseline	Cognitive assessments; Demographic; Genomics; Metabolomics; Imaging; Physical activity	Broad medical and health research
11	OASIS [16]	2000 +	2007-present	USA	Vary by sub-project	18–96	Biomarker; Clinical; Cognitive assessments; Imaging;	Accessible imaging data
12	CLSA [17]	50,000 +	2008-present	Canada	Variable	45–85	Cognitive assessments; Demographic; Lifestyle; Medical History;	Aging
13	BNA [18]	30,000 +	2009-present	France	Variable	Mean age of 76	Clinical; Cognitive Assessments; Demographic;	Alzheimer’s disease
14	TILDA [19]	8,000 +	2009 -present	Ireland	Every 24 months	50 +	Biomarker; Cognitive assessments; Demographic; Lifestyle; Social Engagement;	Aging
15	ALFA [20]	2,500 +	2013-present	Spain	Variable	45–75 at baseline	Biomarker; Cognitive assessments; Demographic; Genomics; Lifestyle; Imaging;	Early pathophysiology of Alzheimer’s disease
16	EMIF-AD MBD [21]	1000 +	2013-2018	Several European countries	Variable	50 + at baseline	Biomarker; Clinical; Demographic; Genomics; Metabolomics; Imaging; Proteomics;	Alzheimer’s disease
17	A4 [22]	5,000 +	2014-2020	USA	Variable	65–85	Clinical; Cognitive assessments; Demographic; Imaging;	Amyloid-related memory problems

This is not an exhaustive list of all datasets available for AD research globally. The datasets were selected based on the following criteria: 1) open-access observational datasets, 2) more than 1,000 participants, and 3) prominent use in ML-dementia models developed over the last 10 years. The list is ordered by the year in which the cohort study began

*Abbreviations*: *A4* Anti-Amyloid Treatment in Asymptomatic Alzheimer's study, *ADNI* Alzheimer’s Disease Neuroimaging Initiative, *AIBL* The Australia Imaging, Biomarker and Lifestyle Study, *ALFA* Alzheimer and Families project, *BLSA* The Baltimore Longitudinal Study of Aging, *BNA* The French National Alzheimer Database, *CLHLS* Chinese Longitudinal Healthy Longevity Survey, *CLSA* The Canadian Longitudinal Study on Aging, *EMIF-AD MBD* European Medical Information Framework for Alzheimer’s Disease Multimodal Biomarker Discovery, *NACC* The National Alzheimer’s Coordinating Center, *OASIS* Open Access Series of Imaging Studies, *ROSMAP* Religious Orders Study and Rush Memory and Aging Project, *TILDA* The Irish Longitudinal Study on Ageing, *WRAP* Wisconsin Registry for Alzheimer’s Prevention

**Table 2:** Types of data commonly used in ML-dementia

**Input data Categories**	**Assessments/Techniques**	**Cost**	**Invasiveness**	**Targets**	**Datasets**^b^
Biopsy	Brain tissue biopsy	AU$1,601.8^a^	High	Amyloid plaques, neurofibrillary tangles, etc	-
Blood/CSF biomarker	Lumbar puncture	AU $109^a^	High	CSF-Aβ & CSF-tau [23], neurofilament light chain, etc	2, 3, 4, 5, 6, 7, 8, 9, 11, 17
	Blood testing (e.g., HRMS [24])	US$200—$500 [25]	Minimum	Neurofilament light chain; plasma Aβ_42_/Aβ_40_ [26], p-tau 181, p-tau231, and p-tau217 [27], etc	2, 3, 5, 8, 9,15, 16
Omics (blood/serum, urine, saliva)	Genomics	US$990 for Alzheimer’s disease, familial, plus APOE panel [28]	None/minimum	APP, APOE, PSEN1, PSEN2, etc	1, 2, 3, 4, 5,7, 10, 15
	Metabolomics	US$100—US$500 [29]	None/minimum	Amino acids, carbohydrates, fatty acids, etc	2, 4, 7,8, 10, 15
	Proteomics	$132 [30]	None/minimum	GFAP [31] and LTBP2 [32] etc	2, 4, 8, 15
	Transcriptomics	AU$200 [33]	None/minimum	MicroRNAs, mRNA levels, etc	2, 5, 10, 15
Cognitive assessments	Neuropsychological evaluation	Vary by healthcare system	None	ADAS-Cog, CDR, MMSE, etc	3, 4, 5, 6, 7, 8, 9, 10, 11, 12, 13, 14, 15, 17
	Questionnaire	Labor cost	None	ECOG and FAQ, etc	
Demographics or clinical data from EHR	Census, observational cohort study, clinical history	Labor cost	None	Age, economic status, education, gender, lifestyle, medical history, race, etc	1, 2, 3, 4, 5, 6,7, 8, 9, 10, 11, 12, 13, 14, 15, 16, 17
Imaging	DTI	US$97 [34]	None	White matter integrity, brain network connectivity, etc.	3, 6, 7, 11
	Retinal imaging	AU$55^a^	None	Network complexity, tortuosity, vessel calibers, etc	14
	CT	AU$342.95^a^	None	Structural images	1
	MRI	AU$426.5^a^	None	Functional MRI: tracking alterations in blood flow linked to neural activity [35] structural MRI: brain anatomy images [36]	3, 4, 6, 8, 11, 15, 17
	PET	AU$605.05^a^	None/moderate	Aβ-PET, FDG-PET, tau-PET [37], etc	2, 6, 7, 8, 9, 11, 17
Physiological monitoring	EEG	AU$358.45^a^	None	Brain waves, electrical activity of the brain, etc	1, 17
	ECG	AU$167.55^a^	None	Electrical activity of the heart, etc	1, 2, 10, 17
Speech and language [38]	Microphones & recording	-	None	Acoustic, prosodic, etc	-

*Abbreviations*: *Aβ* amyloid-beta, *ADAS-Cog* Alzheimer's Disease Assessment Scale—Cognitive Subscale, *APOE* apolipoprotein E, *APP* amyloid precursor protein, *CDR* Clinical Dementia Rating, *CSF* cerebrospinal fluid, *CT* computed tomography, *DTI* diffusion tensor image, *ECG* electrocardiogram, *ECOG* Everyday Cognition Scale, *EEG* electroencephalogram, *EHR* electronic health records, *FAQ* Functional Activities Questionnaire, *FDG-PET* fluorodeoxyglucose positron emission tomography, *GDF15* growth differentiation factor 15, *GFAP* glial fibrillary acidic protein, *HMRS* high-resolution mass spectrometry, *LTBP2* latent transforming growth factor beta binding protein 2, *MMSE* Mini-Mental State Examination, *MRI* magnetic resonance imaging, *PSEN1* presenilin 1, *PSEN2* presenilin 2, *PET* positron emission tomography

^a^prices were obtained from Australia Medicare Benefits Schedule website in June 2024 [39]

^b^refer to Table 1 for dataset number

The development of anti-Aβ monoclonal antibodies, such as donanemab [40] and lecanemab [41], has shown promising results in reducing cognitive decline in early treatment scenarios. This underscores the importance of timely intervention. ML can enhance early detection accuracy and personalized stimulation by determining the most effective timepoint to adminster antibodies in the right patients, thereby maximizing their therapeutic benefits. However, it must be noted that while ML can aid in identifying individuals likely to benefit, our global health systems are not fully equipped to provide these early interventions. Monoclonal antibodies require costly monitoring for brain bleeds, which presents challenges not only in funding the necessary scans but also in accessing scanners within a reasonable distance for patients. A recent study showed that novel biomarkers including microRNAs, metabolites and proteins have been identified using ML approaches [42]. Furthermore, it has been demonstrated that patient-level simulations by ML can predict disease trajectories [43], estimate the likelihood of transitioning from MCI to ADem [44] or even successfully forecast the time-to-event outcomes survival probability for MCI participants [45].

Here we provide a comprehensive overview of ML application in dementia (ML-dementia) using non-technical terms to enhance accessibility to a broad readership. Specifically, we evaluate ML from a historical perspective and discuss typical workflows, successful applications within 5 years and challenges—highlighting the evolving utility of ML in biomedical research to enhance diagnosis and management of dementia.

## Machine learning

### Types of ML

ML includes a variety of algorithms designed to learn from data to meet a predefined goal, such as identifying patterns or making predictions about future states. The model updates its settings or '(hyper-)parameters' based on feedback from performance metrics known as 'loss functions', which assesses the accuracy of the model's predictions compared to actual outcomes. Once the model is optimally trained, it can use real-world data to achieve the predefined task [46]. ML techniques are primarily divided into three categories: unsupervised learning, supervised learning, and reinforcement learning, with the first two being more commonly used in dementia research. These categories are discussed in detail below and their advantages and limitations are summarized in Table 3**.**

**Table 3:** Examples of machine learning models

Subtypes	Model name	Advantages	Limitations
**Supervised learning** [47]			
**Linear models**	Linear regression; Logistic regression; Lasso; Ridge; Elastic net, etc	Simple; Computationally efficient; Suitable for linear relationship	Sensitive to outliers
**Non-linear models**	Support vector machine; k-nearest neighbors	Can capture features with non-linear relationships	Computationally intensive; Possible to overfit
**Tree-based models**	Random forest; Decision trees; eXtreme gradient boosting	Robust to outliers; Can capture non-linear relationships and interactions between features	Possible to overfit; Less interpretable
**Probabilistic models**	Naive bayes; Bayesian linear regression	Can be used on small datasets; Robust to noise	Assumption of independence between features; Prior knowledge in data distribution must be known
**Deep learning models**	Multilayer perceptron; Convolutional neural networks; Recurrent neural networks; Transformers; Attention	Capable of learning complex patterns and relationships; Good for image and speech recognition	Requires large amounts of data; Computationally intensive; Lack of interpretability
**Unsupervised learning** [48]			
**Clustering**	k-Means; Hierarchical clustering	Can discover patterns and groups for unlabelled data	Sensitive to scale of data; Assumes certain data distributions
**Dimensionality reduction**	Principal component analysis; t-SNE	Reduces data complexity	Lack of interpretability
**Reinforcement learning** [49]			
**Model-based**	Dyna-Q; Monte carlo tree search	Efficiency	Suitable only for simpler environments; Effectiveness highly depends on the accuracy of the model
**Model-free (Value-based)**	Q-Learning; State-Action-Reward-State-Action	Flexible; Suitable for more dynamic environment; Less sensitive to the initial conditions and hyperparameters	Data and computationally intensive; May not handle stochastic environments effectively
**Model-free (Policy-based)**	REINFORCE; Deterministic Policy Gradient	Flexible; Supports both discrete and continuous action spaces	Data and computationally intensive; Slow to converge; High variance

#### Supervised learning

Supervised learning explores the relationship between input features and the corresponding target outputs, also known as labels. In dementia research, supervised learning can be further categorized based on the predictive target, for instance, classification tasks dealing with categorical labels (e.g., ADem vs CU), regression tasks handling numerical labels (e.g., Clinical Dementia Rating—Sum of Boxes [CDR-SB] and Mini-Mental State Examination [MMSE]). Once the model is trained, it can then make predictions on unlabelled data of the same input.

#### Unsupervised learning

Unsupervised learning operates on unlabelled data, which focuses on uncovering patterns or relationships without considering any predefined labels. This approach includes 1) clustering tasks such as identifying subtypes of dementia based on biological, neuropsychological, and demographic features and 2) data compression such as using principal component analysis to simplify and summarize complex data.

#### Reinforcement learning

Reinforcement learning (RL) is used to learn and improve decision making by continuously receiving feedback through interaction with external conditions and observing the response. This approach is less commonly used than the supervised and unsupervised methods. RL can be classified as model-free and model-based types; model-free RL operates without a predefined model, while model-based RL is preferred for incorporating domain knowledge (i.e., existing clinical knowledge). RL could mainly be employed to simulate and predict cognitive states, as well as to estimate the probability of transitioning between cognitive states.

### Statistical analysis versus ML approaches

Traditional statistical methods include a hypothesis-driven approach and statical inference (i.e., generalizing findings from a subset of data to a large population). Such approach relies on strong assumptions about the data, e.g., the data follows a normal distribution to fit existing theoretical models [50]. However, these traditional statistical methods often encounter practical challenges in complex real-world scenarios, as the assumptions made may not be satisfied in clinical practice [2]. In contrast, ML adopts a more data-driven approach with minimal assumptions, and it concentrates on prediction rather than inference [2]. However, statistical models and ML techniques sometimes overlap; e.g., both methods often employ linear and logistic regression models to meet statistical goals or to achieve simple linear predictions in ML contexts. It must be noted that ML possesses the capability to process and analyze extensive and complex datasets, such as omics data, effectively uncovering patterns or capturing interactions that might be omitted or overlooked by the traditional statistical analysis [2]. Therefore, ML is often beneficial to clinical research, where data is inherently multidimensional with a diverse array of variables.

### The history and typical workflow of ML techniques in dementia research and clinical applications

Prior to the year 2000, research primarily focused on clarifying the genetic and biochemical foundations of AD, with significant emphasis on the roles of Aβ and familial genetic mutations [51]. In the subsequent decade (2000–2010), scholarly attention shifted towards differentiating AD from CU mostly using ML model such as support vector machines alongside brain imaging techniques [52]. In the following five years or so, researchers focused on predicting clinical progress in MCI patients using multi-kernel support vector machine (SVM, a ML model) with longitudinal data from magnetic resonance imaging (MRI) and positron emission tomography (PET) [53].

**Fig. 1:**
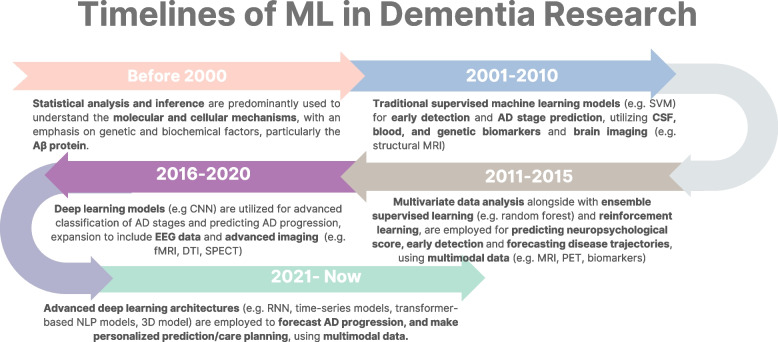
Timelines of ML in dementia research. Aβ = amyloid-beta; AD = Alzheimer’s dementia; CNN = convolutional neural network; CSF = cerebrospinal fluid; DTI = diffusion tensor image; EEG = electroencephalogram; fMRI = functional magnetic resonance imaging; MRI = magnetic resonance imaging; NLP = natural language processing; PET = positron emission tomography; RNN = recurrent neural network; SPECT = single-photon emission computed tomography; SVM = support vector machine. This figure is created using Canva (www.canva.com)

Since then, ML or deep learning, a subset of ML that uses neural network to simulate the learning process of human [54], has been used to classify disease subtypes and stages. Similar to how the human brain employs interconnected neurons for information processing, neural networks in ML use nodes (artificial neurons) and their interconnections to mimic the brain's structure and functionality. This design facilitates pattern recognition and decision-making. For instance, Ramzan et al. [55] utilizes resting-state function MRI with Residual Network architecture to classify AD into: CU, significant memory concern, early-MCI, MCI, late-MCI, and ADem. In more recent years, the adoption of advanced deep learning architectures, such as time-series models has expanded. For example, hybrid deep learning frameworks based on Bidirectional Long Short-Term Memory models leverage multimodal data (i.e., MRI, PET, and neuropsychological evaluation) to enhance the classification of CU and early MCI [56]. A timeline summarizing the use of ML in dementia research is presented in Fig. 1.

**Fig. 2:**
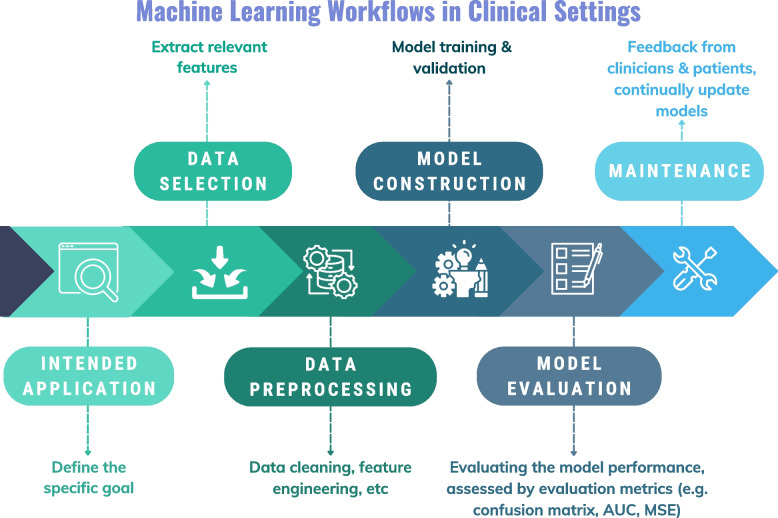
General machine learning model workflows in clinical settings. AUC = area under the curve; MSE = mean squared error. This figure is created using Canva (www.canva.com)

The general workflow to build and apply the ML-dementia model is summarized in Fig. 2, which can be separated into six key steps, including 1) Intended application, 2) Data selection, 3) Data pre-processing, 4) Model Construction, 5) Model evaluation, and 6) Maintenance. We have provided a detailed description for each step in Supplementary Material – ML workflow.

### Data used in ML-dementia studies

Several observational dementia datasets have been used for ML model construction and validation (Table 1), such as the Australian Imaging, Biomarker and Lifestyle (AIBL) study [57] and the Alzheimer's Disease Neuroimaging Initiative (ADNI) study [13]. These datasets are often longitudinal, involving thousands of participants, spanning several decades with regular follow-ups, and some are still actively recruiting. These datasets feature a diverse range of participant demographics, typically focusing on middle-aged adults from various racial, ethnic and educational backgrounds. Each dataset has a distinct focus. For instance, Open Access Series of Imaging Studies [OASIS] [16] concentrate on brain imaging, while the Religious Orders Study and Rush Memory and Aging Project [ROSMAP] [9] aim to understand aging processes. Data collection and testing within the same dataset can vary depending on the project's phases or aims. For example, ADNI adapts its data collection strategies across five phases, and OASIS divides its datasets to address specific research goals. While most datasets listed in Table 1 primarily address AD, others such as the UK Biobank [15] and the Framingham Heart Study [6], provide a broader insight across various health outcomes within larger cohorts.

A variety of data/sample collection methods have been employed in these studies, which can be categorized as per their level of invasiveness (Table 2). Invasive methods, such as cerebrospinal fluid collection through lumbar puncture, are commonly used to obtain biomarkers (Aβ and tau) and markers of neurodegeneration [1]. The AT(N) 2018 framework [58], categorizes the progression of AD into different stages based on specific combinations of these biomarkers (Table 4). Compared to lumbar puncture, venous blood collection is considered less-invasive, and often used for biomarker research and omics (genomics, transcriptomics, proteomics, and metabolomics) analysis [59]. Non-invasive methods such as MRI and PET are employed to study brain structure and Aβ levels [1]. Neuropsychological evaluation (Table 5) are also non-invasive, which are quantitative measures of cognitive functions across various disease stages (Table 6) [60]. Demographic information, lifestyle data and medical history are often self-reported or collected using questionnaires and are used as baseline predictors in the majority of studies [61].

**Table ﻿4:** 2018 NIA-AA research framework [58] for biological definition of Alzheimer’s disease

A	T	N	CU (Cognitively unimpaired)	MCI (Mild Cognitive Impairment)	Dementia
-	-	-	Cognitively unimpaired	MCI not caused by Alzheimer’s disease	Dementia not caused by Alzheimer’s disease
		+			
	+	-			
		+			
+	-	-	Preclinical Alzheimer’s disease	Prodromal Alzheimer’s disease	Alzheimer’s dementia
		+			
	+	-		Alzheimer’s pathologic change with MCI	Alzheimer’s pathologic change with dementia
		+		MCI suspects not caused by Alzheimer’s disease	Dementia suspects not caused by Alzheimer’s disease

A: amyloid-beta levels detected by  PET or cerebrospinal fluid analysis

T: tau pathology evidenced by tangles and PET or cerebrospinal fluid biomarkers

N: neurodegeneration indicated by MRI atrophy, ^18^F-Fluorodeoxyglucose—PET hypometabolism, or high cerebrospinal fluid tau

**Table 5:** Examples of neuropsychological tests for dementia research or clinical diagnosis

Method	Domain	Name of assessments
Questionnaire	Global functioning and behavior	Everyday Cognition (ECOG), Functional Assessment Questionnaire (FAQ), etc
	Psychiatrics	Geriatric Depression Scale (GDS), Neuropsychiatric Inventory (NPI), etc
Neuropsychological evaluation	Global functioning and behavior	Clinical Dementia Rating-Sum of Boxes (CDR), Mini Mental State Examination (MMSE), Montreal Cognitive Assessment (MoCA), etc
	Test battery	Alzheimer's Disease Assessment Scale (ADAS), Cogstate Brief Battery (CBB), etc
	Language	Boston Naming Test (BNT), Rey Auditory Verbal Learning Test (RAVLT), etc
	Memory	Rey Auditory Verbal Learning test (RAVLT); Logical memory IIA Delayed (LOGIMEM), etc
	Visuospatial ability	Clock Drawing Test, Hooper Visual Organization Test, etc
	Recognition and processing speed	Face Recognition Tests, Benton Visual Retention Test, etc
	Executive functioning	Wisconsin Card Sorting Test, Stroop Test, Trail Making Test (Part B), etc
	Emotional and personality assessment	Minnesota Multiphasic Personality Inventory, Beck Depression Inventory, etc

**Table 6:** CDR-SB and MMSE scores for cognitive health classification

**Cognitive health**	**Substage**	**CDR-SB Score (0–18)** [62]	**MMSE Score (0–30)** [63]
CU (cognitive unimpairment)	-	0	30
MCI (mild cognitive impairment)	Questionable impairment	0.5–2.5	26–29
	Very mild dementia	3.0–4.0	
Alzheimer’s dementia	Mild dementia	4.5–9.0	21–25
	Moderate dementia	9.5–15.5	11–20
	Severe dementia	16.0–18.0	0–10

*CDR-SB* Clinical Dementia Rating-Sum of Boxes, *MMSE* Mini-Mental State Examination

## Existing ML-dementia models using non/less-invasive data

**Fig. 3:**
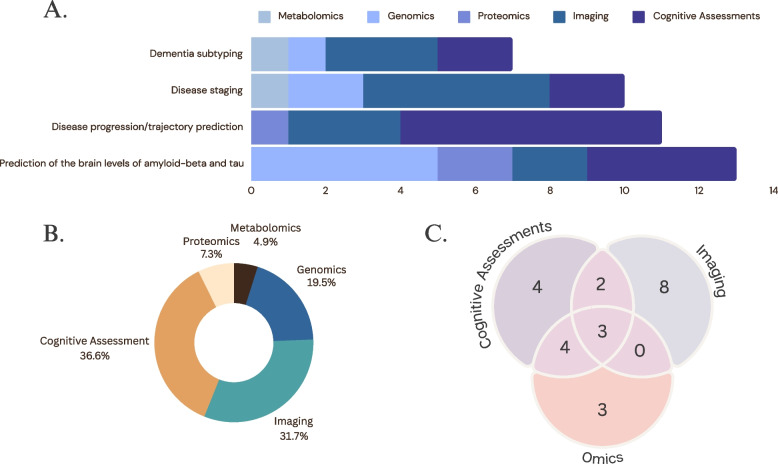
Types of data used in ML models. **A** Counts of various data types used in four major ML-dementia applications; **B** Donut chart showing the distribution of data types used in the selected studies (Table 7); **C** Venn diagram illustrating the overlap of data types used in the selected studies shown in Table 7. This figure is created using Canva (www.canva.com)

The following section reviews ML models using input data collected via non-/moderately invasive approaches. These data include demographics (age, gender, ethnicity, family history), medical history, neuropsychological evaluation, blood (omics, biomarkers), and brain imaging. Studies published between 2019 and 2024 were selected based on uniqueness in methodology, which is summarized in Table 7 and Fig. 3.

**Table 7:** Dementia ML models using non/less-invasive data as input predictors

**A.** **Dementia subtyping**				
**Study**	**Participants**	**Algorithm(s)**	**Predictors**	**Findings/Results**
Castellazzi et al. (2020) [64]	NINCDS2-ARDA criteria (n_ADem_ = 33, n_VAD_ = 27, *n* = 15 for testing)	ANN, SVM, Adaptive Neuro-Fuzzy Inference System	Diffusion tensor imaging and MRI	ADem vs. VaD with AUC = 0.853 using ANFIS algorithm; multimodal input feature set (e.g., DTI + rs-fMRI metrics) have better performance than a unimodal feature set
Nemoto et al. (2021) [65]	The Brain Functions Laboratory, Inc. (n_DLB_ = 101, n_ADem_ = 69, n_CU_ = 38)	RNN	MRI	ADem vs. DLB with ACC = 79.2%
Nguyen et al. (2023) [66]	ADNI2(n_CU_ = 190_,_ n_ADem_ = 149) NIFD(n_CU_ = 136_,_ n_FTD_ = 150) NACC(n_CU_ = 2182, n_ADem_ = 485, n_FTD_ = 37)	Ensemble of 3D U-Nets	MRI	ADem vs. FTD with AUC = 0.938 evaluate in-domain, and AUC = 0.916 evaluating out-of-domain
Qiang et al. (2024) [67]	UK biobank (n_total_ = 274,160; n_ACD_ = 5274; n_ADem_ = 2346; n_VAD_ = 1221)	CPH; LightGBM	Demographics, genetics, plasma metabolome	ACD/ADem/VaD with AUC = 0.857 for ACD; AUC = 0.861 for ADem; AUC = 0.873 for VaD
**B. Disease staging**				
**Study**	**Participants**	**Algorithm(s)**	**Predictors**	**Findings/Results**
Marzban et. al. (2020) [68]	ADNI (n_CU_ = 185; n_MCI_ = 106; n_ADem_ = 115)	CNN	Diffusion tensor imaging and MRI	AUC = 0.84 for MCI/CU; AUC = 0.94 for ADem/CU
Wang et al. (2020) [69]	ROSMAP brain sample (n_CU_ = 51, n_MCI_ = 31; n_ADem_ = 37) serum sample (n_CU_ = 446; n_MCI/ADem_ = 120) serum progression sample (n_CU_ = 356; n_CUp_ = 90)	RF	Plasma metabolome;	CU vs. MCI/ADem with AUC = 0.772 for metabolite level model; health vs. MCI/ADem with AUC = 0.731 for metabolomic pathway level model
Venugopalan et. al. (2021) [70]	ADNI (n_CU_ = 598; n_MCI_ = 699; n_ADem_ = 707)	DT, RF, SVM, KNN for classification; Auto-encoder and 3D CNN for preprocessing	3D MRI images, cognitive assessments, demographics and genetic (single nucleotide polymorphisms)	Single modality for the best performance: ACC_CU_ = 83 ± 7%; ACC_MCI_ = 74 ± 6%; ACC_ADem_ = 85 ± 3% Combine three modalities for the best performance: ACC_CU_ = 88 ± 2%; ACC_MCI_ = 80 ± 2%; ACC_ADem_ = 87 ± 2%
Naz et al. (2022) [71]	ADNI (n_CU_ = 95, n_ADem_ = 95, n_MCI_ = 146)	AlexNet, GoogLeNet, VGG-16/19, ResNet-18/50/101, MobileNetV2, InceptionV3, Inception-ResNet-V2 and DenseNet201	MRI	MCI vs. ADem with AUC = 0.993 CU vs. ADem with AUC = 0.989 CU vs. MCI with AUC = 0.970
Rye et. al. (2022) [72]	ADNI (n_MCIs_ = 357, n_MCIc_ = 321)	Ensemble based model; RF	Cognitive assessments, genetic APOE status, hippocampal volume	MCIs vs. MCIc using RF ACC = 0.746
Hashmi & Barukab (2023) [73]	OASIS (n_CU_ = 639, n_very-mild-ADem_ = 645, n_mild-ADem_ = 662, n_moderate-ADem_ = 624)	Deep learning reinforcement learning for active learning; XGBoosting	MRI	CU: F1-score = 0.82 Very-mild-ADem: F1-score = 0.86 Mild-ADem: F1-score = 0.90 Moderate-ADem: F1-score = 0.94
Mahendran et. al. (2023) [74]	Gene Expression Omnibus data under National Centre for Biotechnology Information (n_ADem_ = 439, n_non-ADem_ = 257) DNA methylation (n_ADem_ = 68, n_non-ADem_ = 74)	Deep Belief Network	Genomic, e.g., DNA methylation and microarray gene expression	ADem vs. non-ADem: ACC = 82%
**C. Disease progression/trajectory prediction**				
**Study**	**Participants**	**Algorithm(s)**	**Predictors**	**Findings/Results**
Beltrán et al. (2020) [75]	ADNI (*n* = 815)	CART, RF, GB & SVM	Cognitive assessments, demographic, and plasma proteomics	MCIs vs. MCIc with AUC = 0.71
Jiang et al. (2020) [76]	ADNI (n_MCIs_ = 165; n_MCIp_ = 137)	Functional ensemble survival tree	Cognitive assessments, demographic	Simulate the trajectory of cognitive assessments; AUC = 0.847 for predicting the AD converter for MCI participants
Kwak et al. (2021) [77]	SMG-SNU Boramae Medical Center (n_CU_ = 285; n_MCI_ = 1057; n_ADem_ = 1300)	LR; SVM	Cognitive assessments	MAE = 0.46 for predicting CDR-SB value for MCI; MAE = 0.39 for predicting CDR-SB for ADem;
Lian et. al. (2021) [78]	ADNI-1 for training (n_CU_ = 226; n_MCIs_ = 225; n_MCIc_ = 165; n_AD_ = 181) ADNI-2 for testing (n_CU_ = 185; n_MCIs_ = 234; n_MCIc_ = 37; n_ADem_ = 143)	Multitask weakly-supervised attention network	MRI	RMSE = 1.501 for CDR-SB, RMSE = 5.701 for ADAS-Cog; RMSE = 2.244 for MMSE
Mofrad et. al. (2021) [79]	ADNI (n_MCIs_ = 333, n_MCIc_ = 333, n_MCIs_ = 333, n_MCIc_ = 333)	Ensemble based model with soft voting strategy	Cognitive assessments, MRI image	MCIs vs. MCIc: ACC = 76 ± 4% for cognitive assessments only; ACC = 77 ± 3.7% adding MRI features; CUs vs. CUc: ACC = 56 ± 6% for cognitive assessments only; ACC = 61 ± 5.7% adding MRI features;
Saboo et al. (2021) [80]	ADNI (n_CU_ = 52; n_AD_ = 23; n_eMCI_ = 58; n_LMCI_ = 27)	Reinforcement learning	Cognitive assessments, demographic, MRI and PET	MAE = 0.537 for predicting MMSE 10-year cognition progression trajectory
Mukherji et. al. (2022) [81]	ADNI (n_CU_ = 200, n_MCI_ = 400, n_ADem_ = 200)	RNN; LSTM; MLP	Cognitive assessments	MCIs vs. MCIc with ACC = 77.88% for predicted cognitive states 3-year after
Bucholc et al. (2023) [82]	NACC-UDS (2-year model: n_MCIs_ = 656; n_MCIp_ = 112; 3-year model: n_MCIs_ = 656; n_MCIp_ = 65;)	LR, SVM, RF	Cognitive assessments	Predict several cognitive assessment score with best performance of ACC = 87.5%
Zou et al. (2023) [83]	ADNI (n_MCIs_ = 601; n_MCIp_ = 330) NACC (n_MCIs_ = 1742; n_MCIp_ = 759)	Multivariate functional mixed model framework	Cognitive assessments, demographic, and genetic APOE status	Simulate the trajectory of cognitive assessments and the time to dementia onset; integrated AUC = 0.839 for Instantaneous model landmark times at 3
**D. Prediction of the brain levels of amyloid-beta and tau**				
**Study**	**Participants**	**Algorithm(s)**	**Predictors**	**Findings/Results**
Palmqvis, et al. (2019) [84]	Train: BioFINDER (*n* = 346) Validation: ADNI (*n* = 170) Test: ADNI (*n* = 661)	LASSO, Logistic Regression	Cognitive assessments, demographic, genetics and plasma proteomics;	Predict Aβ + in PET or CSF*:* Training: AUC = 0.85 with delayed recall mode + plasma Aβ_42_/Aβ_40_ Testing: AUC = 0.83 with delayed recall mode + plasma Aβ_42_/Aβ_40_
Langford et. al. (2020) [85]	A4 (*n* = 1323 for Aβ + ; *n* = 3163 for Aβ-)	XGBoost	Cognitive assessments, demographic and genetic APOE status	Predict Aβ + in PET: AUC = 0.6 for a web-based battery; AUC = 0.74 with APOE and cognitive assessments
Shan et al. (2021) [86]	ADNI (n_SMC_ = 170; n_eMCI_ = 317; n_LMCI_ = 236)	KNN, DT, SVM, RF	Clinical, cognitive assessments, demographic and genetic APOE status	Predict Aβ + in PET for 6 separate groups with the best ACC = 90.4% for the SMC male group
Janelidze et al. (2021) [87]	BioFINDER (n_CU_ = 182; n_MCI_ = 104) ADNI (*n* = 59 for Aβ + ; *n* = 63 for Aβ-)	Logistic regression	Plasma biomarker	Predict Aβ status: AUC = 0.86 in CSF; AUC = 0.83 in PET With immunoprecipitation-coupled mass spectrometry developed at Washington University achieve the best in all 8 assays
Lew et. al. (2023) [88]	ADNI (*n* = 1027 for amyloid; *n* = 375 for Tau; *n* = 1239 for fluorodeoxyglucose)	logistic regression for binary classification	Cognitive assessment, demographic, genetic APOE status and MRI	AUC = 0.79 for amyloid-beta; AUC = 0.73 for tau; AUC = 0.86 for neurodegeneration
Zhang et. al. (2022) [89]	European Medical Information Framework for Alzheimer’s Disease Multimodal Biomarker Discovery (n_CU_ = 311; n_AD_ = 184; n_MCI_ = 386)	7-layer neural network	Demographic, genetic APOE status and proteomics	AUC = 0.782 for Amyloid, AUC = 0.674 for p-tau, and AUC = 0.734 for t-tau. AUC = 0.831for A + T + N + vs. A-T-N-

*Abbreviations*: *A4* Anti-Amyloid Treatment in Asymptomatic Alzheimer’s disease, *ADNI* Alzheimer’s disease Neuroimaging Initiative, *NACC-UDS* National Alzheimer’s coordinating center uniform data set, *NIFD* National institute of Fashion Designing, *NINCDS-ADRDA* National Institute of Neurological and Communicative Disorders and Stroke and the Alzheimer’s Disease and Related Disorders Association, *ROSMAP* Religious Orders Study and Rush memory and Ageing; *SMG-SNU*  Seoul Metropolitan Government – Seoul National University, *ACC* accuracy, *Aβ* + amyloid-beta positive, *Aβ-* amyloid-beta negative, *ACD* all-cause dementia, *ADem* Alzheimer’s dementia, *AFT* accelerated failure time model, *ANN* artificial neural network, *APOE* apolipoprotein E, *AUC* area under the receiver operating characteristic curve, *CART* category and regression tree, *CPH* cox proportional hazard regression model, *CSF* cerebrospinal fluid, *CU* cognitive unimpairment, *CUs* cognitive unimpairment stable (i.e., no progression to MCI or AD), *Cup* cognitive unimpairment progression (i.e., progression from CU to MCI/AD), *DLB* dementia with Lewy bodies, *DT* decision tree, *eMCI* early mild cognitive impairment, *GB* gradient boosting, *KNN* k-nearest neighbour, *LASSO* least absolute shrinkage and selection operator, *LightGBM* light gradient boosting machine, *LMCI* late mild cognitive impairment, *LR* linear regression, *LSTM* long short-term memory, *MAE* mean absolute error, *MCI* mild cognitive impairments, *MCIs* mild cognitive impairment stable, *MCIp* mild cognitive impairments progression to Alzheimer’s dementia, *MLP* multilayer perceptron, *MMSE* Mini Mental State Examination, *PET* positron emission tomography, *RF* random forest, *RNN* recurrent neural networks, *SMC* significant memory concern, *SVM* support vector machine, *VaD* vascular dementia, *XGBoosting* eXtreme gradient boosting

### Dementia subtyping

AD is the major cause of dementia, followed by vascular dementia, frontotemporal dementia, and dementia with Lewy bodies [90]. Accurate differential diagnosis is important for clinicians to offer the most suitable care options to the patients [91]. Recent studies utilizing ML and deep learning models have shown relative high accuracy in differential diagnoses by incorporating metabolomics [67] and neuroimaging [64–66] (Table 7A). For instance, Qiang et al. [67] established the associations between 249 metabolites and type of dementia (all-cause dementia, ADem, and vascular dementia) using UK Biobank data. The study employed Cox proportional hazard models and light gradient boosting machine algorithms to generate a metabolic risk score. This score when combined with demographic and neuropsychological test scores achieved an AUC of 0.85 (AUC approaching 1 indicates excellence in discrimination) for the classification of different types of dementia. By employing neuroimaging data, Castellazzi et al. [92] used the adaptive neuro-fuzzy inference systems to distinguish between ADem and vascular dementia. This achieved over 84% accuracy using a combination of features from resting-state functional MRI and diffusion tensor imaging. Moreover, another independent research group [65] achieved ~ 80% accuracy in differentiating dementia with Lewy bodies from ADem using structural MRI data and a residual neural network. Finally, Nguyen et al. [66] introduced an innovative approach, by integrating 3D U-Nets with a multi-layer perceptron classifier to discern ADem from frontotemporal dementia through structural MRI images, attaining an AUC of 0.94.

Although these studies achieved high diagnostic accuracies (~ 80%), only Nguyen et al. [66] validated their model using an external dataset. This raises concerns about the generalizability of these findings and suggests that potential cohort bias cannot be ruled out. It is crucial to further validate these models prior to clinical trial and implementation. Moreover, these studies appear to focus on the differential diagnosis between vascular dementia and ADem (Qiang et al. [67] and Catellazzi et al. [92]) and between frontotemporal dementia and ADem (Nguyen et al. [66]). Future research could explore the possibility of differentiating multiple subtypes of dementia using a single model. Furthermore, all these studies, except Qiang et al. [67], leveraged advanced imaging techniques to capture intricate details of the brain. The reliance on high-resolution imaging data necessitates substantial resources, making it challenging to implement the new technology in clinics.

### Disease staging

Predicting disease stages using either a binary classification (CU vs ADem, CU vs MCI + ADem, CU vs MCI, MCI vs ADem) or CU/MCI/ADem classification is commonly used in ML-dementia. These typically employ omics data [69, 74], neuropsychological evaluation [70], and neuroimaging [68, 70, 71] (Table 7B). Mahendran et al. [74] demonstrated that deep belief network-based approach (accuracy 82%) outperformed SVM (accuracy 78%) and Naïve Bayes (accuracy 76%) in binary classification of CU and ADem using their multi-omics data. In another study, Wang et al. [69] utilized six differentially expressed metabolites, three metabolic pathways and a random forest model to differentiate the MCI + ADem group from CU, and they achieved an AUC of 0.77. MRI data have also been employed to facilitate disease classification. For instance, Naz et al. utilized only structural MRI data [71], and achieved a classification accuracy of 99.27, 98.89 and 97.06% for MCI/ADem, ADem/CU, and MCI/CU, respectively. To generate more complex models, multimodal data (e.g., demographic, medical history, brain volume, neuropsychological evaluation and genetics) have been integrated, such as convolutional neural network model for disease stage classification. For example, using multimodality, Venugopalan et al. [70] achieved a classification accuracy of 83% for CU, 74% for MCI and 85% for ADem.

We noted that model development in most of these studies were challenged by an imbalanced dataset, with AD and MCI often being underrepresented compared to CU individuals due to disease prevalence. Interestingly, Naz et al. [71] manually balanced the dataset by eliminating some of the CU participant data (CU = 95, MCI = 146, ADem = 95). However, this approach reduces the overall dataset size, possibly leading to the model not capturing all critical features for accurate classification [93]. Model overfitting is also expected from using such a small dataset [94]. Future studies could focus on enriching AD and MCI participant data; however, this is currently less practical due to a lack of harmonized datasets that allows data pooling. An alternative approach is to intentionally recruit MCI and ADem participants, as done by Kwak et al. [77]; however, these data may be less suitable for studying the onset and progression of AD. Another major issue is that the classification accuracy is usually less satisfactory for differentiating MCI from AD, as has been reported by Wang et al. [69] and Naz et al. [71]. Using multimodal data could be a potential solution [70], nonetheless, future studies are required to confirm whether their observations are dataset dependent.

### Disease progression/trajectory prediction

The prediction of future disease states or neuropsychological outcomes can be achieved using classification and regression models, as well as simulating disease trajectories using more complex deep learning models (Table 7C). Most classification models categorize MCI-to-dementia progressors and non-progressors. For example, Rye et.al. [72] achieved a 75% of accuracy in predicting whether MCI participants progress to dementia using a random forest model, where neuropsychological evaluation, hippocampal volume and Apolipoprotein E (APOE) genotype were used as input features. An ensemble model was employed by Mofrad et al. [79] for such prediction, where MRI and neuropsychological evaluation were used to achieve a 77% accuracy. Regression models often employ neuropsychological evaluation, such as CDR-SB, ADAS-Cog, and MMSE [77, 78, 82], to estimate disease severity over time. For example, Lian et al. [78] employed a multitask weakly-supervised Attention Network, which is a regression model that built on structural MRI data collected from CU, MCI progressor, MCI non-progressor, and ADem participants to predict 3-year future CDR-SB, ADAS-Cog, and MMSE scores. This model has achieved promising results, with a root-mean-squared error of 1.5, 5.7, and 2.2 for each score, respectively.

For disease trajectory simulation, Bucholc et al. [82] has combined unsupervised and supervised learning techniques, where participants were categorized by their cognitive score trajectories (stable vs deterioration over 2–3 years). The trajectories of each category were then analyzed using random forest, support vector machine, and linear regression (supervised). This approach achieved a ~ 90% accuracy in predicting seven different neuropsychological test scores over 1-year and 2-year intervals, from the correspondent baseline scores. A more complex model, Long Short-Term Memory Recurrent Neural Networks, was used by Mukherji et al. [81] to simulate the trajectory for five neuropsychological tests. This model achieved a prediction accuracy of 85 and 83% for 2-year and 4-year, respectively. Recent work has also focused on dynamically predicting the risk of dementia onset. This is typically achieved using a Cox model, combined with functional data analysis to model longitudinal neuropsychological outcomes. For example, Jiang et al. [76] utilized the functional ensemble random survival forest to characterize the joint effects of neuropsychological evaluation in predicting disease progression, specifically to predict the time to AD conversion in individuals with MCI and to provide personalized dynamic predictions. This approach achieved an AUC of approximately 0.90 over an average follow-up period of 31 months. Similarly, Zou et al. [83] proposed a multivariate functional mixed model framework to simultaneously model multiple longitudinal neuropsychological outcomes and the time to dementia onset, achieving an integrated AUC of over 0.80, with the mean time to visit being 1.12 years.

Mukherji et al. [81], Bucholc et al. [82] and Lian et al. [78] predict disease progression over a fixed interval, while Jiang et al. [76] and Zou et al. [83] simulate disease progression. It should be noted that simulation methods introduce higher variance and complexity compared to fixed interval models [95]; however, they can predict disease status at any time point, whereas fixed interval models can only predict disease status at the end of the interval. Different models may suit varying clinical needs or patient expectations, each balancing its own advantages and limitations. In addition, these complex models are prone to overfitting [94], capturing noise that does not generalize to unseen data. This issue could be exacerbated in studies where the training datasets are relatively small, such as that for Jiang et al. [76] (165 MCI stable, 137 MCI progressor). We have also noted that most of these models, except Lian et al. [78], involve various neuropsychological tests, which often differ between studies. This makes it challenging for external validation and comparison between different models. Future studies should consider developing models based on neuropsychological tests that are routinely used in clinics for easier evaluation, validation and potential implementation.

### Predicting Aβ and tau levels in the brain

ML models have shown promise in predicting AD biomarkers with reasonable accuracy (Table 7D). For predicting Aβ and p-tau levels in the brain, the problem is often simplified into a binary classification, e.g., normal vs high or negative vs positive. Langford et al. [85] employed the extreme gradient boosting algorithm, a scalable tree boosting model to predict Aβ PET positivity (standardized uptake values ≥ 1.15) from demographics (age, education, gender and family history), four neuropsychological tests and APOE genotype., An AUC of 0.74 was achieved. Palmqvist et al. [84] used plasma Aβ_42_/ Aβ_40_ ratios, APOE genotype, and neuropsychological tests for a logistic regression with a lasso penalty model, and achieved an AUC of 0.83. In contrast, Lew et. al. [88] employed a logistic regression model for binary prediction of PET results (high versus low Aβ or p-tau) using MRI and other data (e.g., demographic, APOE genotype, neuropsychological tests and hippocampal volumes etc.). This resulted in an AUC of 0.79 for Aβ and 0.73 for p-tau. Using a seven-layer neural network, 3,635 plasma proteins, age and APOE genotype for the same prediction, Zhang et al. [89] achieved a lower AUC score for Aβ (AUC = 0.78) and p-tau (AUC = 0.67). Their performance is relatively lower than the other studies, which could possibly be due to high feature-to-sample ratio (3000 proteins in 800 participants), which can complicate model training and validation.

Notably, a universally accepted threshold to determine binary classification is lacking. For example, Langford et al. [85] used a threshold of 1.15, while Palmqvist et al. [84] adopted a threshold of 0.738. Whether this would have impacted the prediction performance of the model is unclear. Future studies should consider standardizing this threshold to enable comparisons between models. Another issue with these studies is that the datasets used for model training are relatively small (e.g., 300 participants for Palmqvist et al. [84] and 800 participants for Zhang et al. [89]), possibly due to cost constraints associated with PET and MRI. Research funding bodies could play a role in encouraging (inter)national collaboration and data sharing, as well as endorsing standard data formats (especially for those high-cost experiments) to increase the size of datasets for more robust results.

## Challenges and future directions

ML has been applied to clinical data analysis for more than two decades, and its widespread adoption in clinical research and healthcare has noticeably accelerated. This section will discuss the technical barriers, and the anticipated challenges and potential solutions to applying ML in clinical practice for dementia (summarized in Table 8).

**Table 8:** Challenges, solutions and future directions

Challenges	Solutions/future directions
Missing data	Utilize data imputation technique like mean imputation, multiple imputation by chained equations, etc
Data imbalance	Utilize resampling techniques like Synthetic Minority Over-sampling Technique, etc
Diagnostics error	Expand the use of subjective diagnostics criteria
Non-uniform longitudinal data	Data harmonization
Lack of generalizability	Develop global criteria that balance scientific rigor and practical feasibility
Exclusion of diverse populations	Encourage global collaborative efforts among researchers, clinicians, and regulatory bodies, strategic recruitment of people from culturally and linguistically diverse background
Computational burdens	Utilize efficient algorithm design, high-performance computing resources, and distributed computing platforms
Patient acceptance	Increase public awareness, ensure data transparency, security, and provide psychological support
Clinician acceptance	Offer ML training to medical students and clinicians, develop explainable AI techniques, and involve clinicians in co-design of ML tools to enhance usability and trust
Lack of interpretation for ML-dementia applications	Implement and promote explainable AI techniques like LIME and SHAP to make ML decision-making transparent
Ethical and regulatory considerations	Advocate for local and international ethical guidelines and regulatory compliance, ensure continuous monitoring post-deployment

### Clinical data quality

Given the complex set up of longitudinal studies and heterogenous disease pathology, missing values, outliers, data imbalance are inevitable. Missing data is often due to incomplete responses, data collection errors, technical issues and participant withdrawal [96]. Data scientists either disregard participants with missing data or use imputation techniques (e.g., mean imputation, multiple imputation by chained equations, etc. [97]). Outliers normally result from errors from record, measurement or misclassification. Statistic techniques, such as *z*-scores and interquartile range or box plot are used to detect outliers. Once identified, common approaches involve removing outliers, adjusting into specific percentile, or applying transformations to reduce the skewness of the data distribution [98]. Data imbalance is a commonly encountered issue for dementia dataset, as MCI and ADem occur in a smaller population compared to CU. When MCI/ADem cases are significantly underrepresented compared to CU, it can lead to a biased model performance, where ML models trained on imbalanced data may prioritize the majority and struggle to accurately predict the minority [99]. To address this issue, resampling techniques such as Synthetic Minority Over-sampling Technique [100] can be employed.

The quality of clinical data used to train ML models directly impacts the soundness of the model. The diagnoses are performed by clinicians and neuropsychologists [101, 102], which can sometimes introduce human errors into the dataset. This is because diagnosis is complicated by that 1) preclinical AD is difficult to detect [103], 2) MCI can be misclassified [104], and 3) vascular dementia, Lewy body dementia, and frontotemporal dementia are sometimes misdiagnosed as ADem [105]. Moreover, some neuropsychological tests are influenced by practice effects [106] (repeated testing can artificially improve performance over time), and education background [107] (poor performance for individuals who are less educated), potentially skewing results. Furthermore, the trajectory of dementia varies significantly among individuals due to the complex interplays of age, genetics, sex, and other comorbidities [108]. Some individuals may experience a gradual decline in cognition over many years, while others show rapid deterioration. Many longitudinal studies employ an "up-to-interval" method [75], classifying participants into CU, MCI, ADem, and non-ADem within a specified follow-up period. However, this approach often falls short in capturing the disease trajectory of individuals experiencing gradual cognitive decline. In addition, older participants are more likely to withdraw from the study due to their dependency on others (e.g., reduced mobility discourage their participation), leading to their disease trajectory not fully captured. Cohort study designs can be enhanced to improve data quality. Longitudinal study designs should consider incorporating more objective diagnostic criteria, such as expanding the use of Aβ PET scans, and integration of blood-based biomarkers, tau, and neuroinflammation markers, to enhance the assessments accuracy. Additionally, developing strategies to prolong study follow-up duration is crucial for capturing the full progression of disease states over time. Research funding bodies could play a crucial role in driving this progress by prioritizing investment and providing support to longitudinal studies.

### Data standardization

The existing longitudinal datasets exhibit a lack of uniformity and standardized approach in sample/data collection and record format, making it difficult to validate and compare metrics like accuracy, sensitivity, and specificity between ML models that built on different datasets [109]. For example, although AIBL and ROSMAP collected depression related data, yet different scales were used—AIBL adapted the Hospital Anxiety and Depression Scale while ROSMAP used the Center for Epidemiological Studies Depression scale. The lack of uniformity in data collection could also be attributed to the intrinsic nature of the technology. For example, various platforms, techniques, and environmental factors could introduce biases and variabilities into omics dataset [110]. In addition, omics data is often noisy and sparse, especially when detecting molecules of low abundance, and therefore more prone to batch effect. Furthermore, different annotation systems or reference databases used to identify proteins, metabolites, and genes can lead to mismatches and inconsistencies. Also, different omics dataset may lack of common features due to experiment set up. All these make it less practical to standardize the omics data.

To enhance the performance of ML models in dementia research, addressing variability in data collection methods is crucial. The Alzheimer's Dementia Onset and Progression in International Cohorts initiative [111] exemplifies the successful application of data harmonization, integrating data from five international dementia cohort studies, including the Adult Children Study, ADNI, AIBL, the Dominantly Inherited Alzheimer Network, and the National Alzheimer's Coordinating Center. Similar initiatives should be encouraged, as they are crucial for enhancing statistical power, and enabling more robust ML applications in dementia, leveraging the existing longitudinal datasets. In addition, publication of sample collection protocols, along with raising awareness of the requirements and benefits of data pooling for ML among biomedical and clinician scientists, could promote consistent data collection practices and enhance collaborative research efforts globally. Of paramount importance, inconsistencies in data formats can undermine the effectiveness of ML models. Advanced tools like 'dtool' provide practical solutions for standardizing data formats and enhancing quality by encapsulating data and metadata into consistent, unified dataset structures with readily accessible metadata for both the collective dataset and its individual files [112]. Data repositories could endorse guidelines that only accept datasets meeting standardized criteria.

### Data generalizability

A longitudinal dataset may lack of generalizability. The study setting and enrolment criteria would exclude certain populations based on ethnicity, education level, socio-economic status, or comorbid conditions. For example, research studies might exclude participants with severe cardiovascular diseases or advanced diabetes, arguing that these conditions could confound the cognitive assessments used to diagnose and track ADem progression [113]. Moreover, studies that require participants to be English-speaking exclude individuals from a culturally and linguistically diverse background (e.g., the indigenous population in Australia, who have a higher risk of ADem). These exclusions can result in datasets that fail to fully represent the diverse population affected by dementia. The clinical application of ML models built from biased data will consequently be limited. Collaborative efforts between researchers, clinicians, and regulatory bodies are crucial in developing criteria that balance scientific rigor with practical feasibility. Furthermore, the major dementia longitudinal studies are often restricted to national boundaries, constraining their generalizability and the assessment of their performance in more border real-world scenarios. Researchers are encouraged to employ multiple datasets, where the model is trained on one dataset (e.g., ADNI) and validated on another dataset (e.g., AIBL) [114] to address this challenge.

### Computational and memory burden

Computational and memory burden is another technical challenge to ML-dementia, particularly as recent studies focus on high-dimensional longitudinal omics data. Advanced tools such as the versatile toolbox MEFISTO [115] and the PALMO platform [116] are now capable of modelling spatial and temporal omics data. These tools utilize high-performance computing resources and implement various optimization strategies to improve processing efficiency. However, the high computational and memory demands of these algorithms can limit their applicability in AD studies that involve large sample sizes. Furthermore, the high volume of data requires a robust data management solution. Distributed computing platforms, like Apache Hadoop [117], can be employed to efficiently handle, store, and share the large-scale data, facilitating collaborative efforts across different research groups and locations. However, these platforms are not always affordable, creating a technical barrier.

### From bench to clinic

Artificial intelligence (AI), such as ML, has already demonstrated success in disease tracking, as evidenced by FDA-approved devices like Apple's Atrial Fibrillation History Feature [5]. While ML applications have yet to be implemented in dementia clinical practice, anticipated challenges must be considered for future implementation in dementia diagnosis and care.

#### Acceptance of ML tools by patients

The targeted population for ML-dementia tools is older adults, which raises questions about their readiness to accept these technological innovations [118]. Many older adults are not as technologically adept as younger generations, making it challenging for them to understand ML and its potential in diagnosing and managing diseases. This lack of understanding can result in low trust in ML-generated results, leading to hesitation in their use for healthcare purposes. Moreover, some ML tools collect data using wearable devices, raising privacy concerns among older adults who may be unsure how their data will be used. Furthermore, not all older adults want to receive predictions about their disease progression or early detection due to psychological fears and anxieties [119].

To address these challenges and improve acceptance among older adults, several steps should be taken. Increasing public awareness of ML and its benefits in healthcare is crucial, as many people may not realize that AI/ML are already being used. Ensuring transparency in data usage and robust data security measures can help build trust, while offering a personalized approach where individuals can opt in or out of predictive analyses can promote autonomy [120]. Providing comprehensive psychological support can help individuals cope with the emotional impact of potential diagnoses and empower them to make informed decisions about their health and care plans. By addressing these concerns through patient education, demonstrating the reliability and benefits of ML tools, and ensuring robust data security measures, we can foster greater acceptance of ML-dementia tools among older adults.

#### Acceptance of ML tools by clinicians

Clinicians tend to prefer techniques that are transparent and interpretable, aligning with conventional clinical reasoning. One of the barriers for clinicians to trust and uptake the output of ML models is the opaque nature of these algorithms, often referred to as "black boxes." ML models can obscure the logic behind their complex decision-making processes, sometimes producing results that cannot be easily justified by existing biomedical knowledge. The "black box" nature of ML potentially erodes clinicians' trust, hindering the adoption of these models in clinical practice. In response to these challenges, there is an increasing focus on developing explainable AI techniques, such as Local Interpretable Model-agnostic Explanations (LIME) and SHapley Additive exPlanations (SHAP) [121]. These methods aim to make the decision-making processes of ML models more transparent and understandable, thereby can potentially enhance trust among clinicians. Another significant challenge is that many clinicians have not received formal training in ML, which can hinder their ability to effectively use and explain these tools to patients [122]. Providing basic education about ML to clinicians and incorporating an AI/ML training component in medical school curriculum can enhance their ability to use innovative tools and communicate the benefits to patients. Of paramount importance, involving clinicians in the co-design of ML-dementia models can ensure AI/ML tools meet clinical needs and foster greater acceptance and integration into practice. Last but not least, some clinicians are hesitant to accept AI/ML tools due to concerns about job displacement [122]. However, it is essential to understand that AI/ML tools are designed to augment, not replace, the work of clinicians, similar to other diagnostic tests. Clinicians should be assured that their clinical judgment cannot be replaced by AI/ML and that the role of AI/ML in clinical practice should be clearly defined in relevant guidelines.

#### Ethics and regulatory considerations

The integration of AI/ML in healthcare brings forth numerous ethical and regulatory concerns that could potentially impede their implementation. Recently, the World Health Organization issued new guidance on the ethics and governance of AI technology applications in healthcare [123], emphasizing the need for AI/ML developers to prioritize ethical principles. To facilitate the potential implementation of AI/ML tools in dementia diagnosis and management, we also advocate for the development of local guidelines to fit the culture/religious needs. On the regulatory front, compliance with healthcare regulations is indispensable. Regulatory bodies, such as FDA, the European Medicines Agency, and the Therapeutic Goods administration (Australia), should get prepared for processing more applications for AI/ML medical devices in the future. A clear approach must be established for post-deployment continuous monitoring and reporting, to maintain their safety and effectiveness in the clinic [122]. More importantly, it is crucial that regulations should clearly define the responsibilities and accountabilities of AI/ML developers and healthcare providers for any errors generated by AI/ML tools. This includes specifying the extent of liability for developers in the event of AI/ML malfunction or incorrect predictions, as well as outlining the role of healthcare providers in interpretating AI/ML outputs before making clinical decisions. Regulations should also detail mechanisms for reporting and addressing errors, as well as protocols for updating and improving AI/ML tools from reported errors. An in-depth discussion on regulatory matters concerning ML/AI is outside the scope of this review. Regulatory bodies, clinicians, and public health experts are encouraged to work on regulatory matters to prepare our healthcare systems for the implementation of AI/ML tools.

### Supplementary Information

Supplementary Material 1.

## Data Availability

No datasets were generated or analysed during the current study.

## Abbreviations

Aβ: Amyloid-beta
AD: Alzheimer’s disease
ADem: Alzheimer’s dementia
ADAS: Alzheimer's Disease Assessment Scale—Cognitive Subscale
ADNI: Alzheimer's Disease Neuroimaging Initiative
APOE: Apolipoprotein E
AI: Artificial intelligence
AIBL: Australian Imaging, Biomarker and Lifestyle (Study)
AUC: Area under the curve
CDR-SB: Clinical Dementia Rating—Sum of Boxes
CU: Cognitive unimpaired
MCI: Mild cognitive impairment
ML: Machine learning
MMSE: Mini-Mental State Examination
MRI: Magnetic resonance imaging
OASIS: Open Access Series of Imaging Studies
PET: Positron emission tomography
RL: Reinforcement learning
ROSMAP: Religious Orders Study/Memory and Aging Project
SVM: Support vector machine
