# Supplementary material for: Understanding machine learning applications in dementia research and clinical practice: a review for biomedical scientists and clinicians
Source: Alzheimers Res Ther. 2024 Aug 1;16:175. doi: 10.1186/s13195-024-01540-6 (PMC11293066; doi:10.1186/s13195-024-01540-6)
Supplement: Supplementary file 1 — Supplementary Material 1. [file 13195_2024_1540_MOESM1_ESM.docx]

**ML-Dementia study workflow** – a mini tutorial for biomedical scientists and clinicians

The workflow to build and apply the ML-dementia model**,** which can be separated into six key steps.

**1. Intended application.**

The initial and most crucial step before modeling is to define the intended clinical application. Currently, the major clinical applications of ML-dementia include identifying biomarkers and risk factors, classifying disease stages, estimating an individual's age of ADem onset, predicting disease progression, and drug discovery [1].

**2. Data selection**

Data required by predetermined research objectives are extracted from a database, such as the AIBL study and the ADNI study. Categorical and numerical data should be selected as per the intended ML model(s) to be used, and data that contains >50% missing value should be avoided [2].

**3. Data pre-processing**

For longitudinal data, data collected from a single participant at different collection timepoints should be carefully compiled against their unique participant IDs. The preprocessing stage is a fundamental step ensuring the data used for model construction is clean and valid. Clinical data often contain missing values due to factors like data recording errors or participant withdrawal [3], making it essential to address this issue either by omitting incomplete patient records [4] or by employing sophisticated imputation techniques such as Multiple Imputation by Chained Equations [5]. This stage also includes feature engineering, which involves dimension reduction [6] (e.g., principal component analysis) and feature selection [7] (e.g., minimum redundancy maximum relevance) to enhance model performance and mitigate multicollinearity, thus providing robust result. In addition, normalization is necessary to ensure that all data are on the same scale, further refining the dataset for analysis and model construction [8].

**4. Model construction**

Model construction involves selection of algorithm(s) that meet the needs of the intended clinical application, followed by model training and validation. A portion of the dataset is usually used for model training, where the parameters of the model are adjusted to best represent the underlying patterns. The remaining data can be used for validation, which is designed to detect whether the model can generalize properly from the training data to unseen data, namely overfitting or underfitting, and to facilitate the fine-tuning of specific model hyperparameters [9].

**5. Model evaluation**

Evaluation is ideally performed in an independent external dataset [10], where the model is deployed for a specific target based on unseen data with same structure as the training and validation data. For supervised learning, the choice of evaluation metrics depends on whether the model is used for classification (area under the receiver operating characteristic curve [AUC], accuracy, confusion matrix) or regression tasks (mean squared error [MSE], mean absolute error [MAE], and R-square). For unsupervised learning, evaluation metrics such as the Silhouette Coefficient and Dunn Index are used to assess clustering quality [11]. For reinforcement learning, evaluation often considers the total cumulative reward, learning stability and the model generalisability [12].

**6. Maintenance**

Model developers are required to actively incorporate feedback from clinicians and patients [13]. This is important for the refinement of the model, improving utilization, user acceptability and large-scale clinical use [14].  The model should be continuously updated with new data and research findings to keep it current and robust.

**REFERENCES**

1. Tsoi KK, Jia P, Dowling NM, Titiner JR, Wagner M, Capuano AW, et al. Applications of artificial intelligence in dementia research. Camb Prisms Precis Med. 2023;1:e9.

2. Marshall A, Altman DG, Royston P, Holder RL. Comparison of techniques for handling missing covariate data within prognostic modelling studies: a simulation study. BMC Med Res Methodol. 2010;10:1–16.

3. Heymans MW, Twisk JWR. Handling missing data in clinical research. J Clin Epidemiol. 2022;151:185–8.

4. Lu L, Wang H, Elbeleidy S, Nie F. Predicting cognitive declines using longitudinally enriched representations for imaging biomarkers. IEEE Trans Med Imaging. 2021;40;891-904.

5. Kato R, Hoshino T. Semiparametric Bayesian multiple imputation for regression models with missing mixed continuous–discrete covariates. Ann Inst Stat Math. 2020;72:803–25.

6. Li S, Yang P, Lanfranchi V. Examing and Evaluating Dimension Reduction Algorithms for Classifying Alzheimer’s Diseases using Gene Expression Data, in 2021 17th International Conference on Mobility, Sensing and Networking (MSN). IEEE, 2021.

7. Wang G, Lauri F, El Hassani AH. Feature selection by mRMR method for heart disease diagnosis. IEEE. 2022;10:100786–96.

8. Kang M, Tian J. Machine learning: data pre‐processing., In Prognostics and Health Management of Electronics. John Wiley and Sons;2018.

9. Raschka S. Model evaluation, model selection, and algorithm selection in machine learning. ArXiv Prepr ArXiv181112808. 2018.

10. Cabitza F, Campagner A, Soares F, de Guadiana-Romualdo LG, Challa F, Sulejmani A, et al. The importance of being external. methodological insights for the external validation of machine learning models in medicine. Comput Methods Programs Biomed. 2021;208:106288.

11. Palacio-Niño J-O, Berzal F. Evaluation metrics for unsupervised learning algorithms. ArXiv Prepr ArXiv190505667. 2019.

12. Khetarpal K, Riemer M, Rish I, Precup D. Towards continual reinforcement learning: A review and perspectives. J Artif Intell Res. 2022;75:1401–76.

13. Sapyta J, Riemer M, Bickman L. Feedback to clinicians: theory, research, and practice. J Clin Psychol. 2005;61:145–53.

14. Kumah E, Ankomah SE, Kesse FO. The impact of patient feedback on clinical practice. Br J Hosp Med. 2018;79:700–3.
